# The impact of health literacy interventions on glycemic control and self‐management outcomes among type 2 diabetes mellitus: A systematic review

**DOI:** 10.1111/1753-0407.13436

**Published:** 2023-07-05

**Authors:** Jamila Butayeva, Zubair Ahmed Ratan, Sue Downie, Hassan Hosseinzadeh

**Affiliations:** ^1^ School of Health & Society, Faculty of the Arts, Social Sciences and Humanities University of Wollongong Wollongong New South Wales Australia; ^2^ Discipline of Medical and Exercise Science, Faculty of Science, Medicine and Health, School of Medicine University of Wollongong Wollongong New South Wales Australia

**Keywords:** diabetes mellitus type 2, glucose control and clinical trials, health literacy, self‐management, 2型糖尿病, 血糖控制和临床试验, 健康素养, 自我管理

## Abstract

Diabetes imposes an increasing health and economic burden on individuals living with it and their societies worldwide. Glycemic control is necessary to reduce morbidity and mortality of type 2 diabetes mellitus (T2DM). Self‐management is the primary tool for managing diabetes. Health literacy (HL) is the primary driver of self‐management activities. The aim of this review is to evaluate the impact of HL interventions on glycemic control and self‐management outcomes among T2DM. MEDLINE, CINAHL, PubMed, Cochrane, Scopus, and Web of Science were searched for eligible papers. Fifteen randomized controlled trials published in English between 1997 and 2021, used HL‐driven intervention, and measured the level of glycohemoglobin A1c (HbA1c) and self‐management of T2DM patients were included in this review. The findings showed that HL‐driven intervention had a positive impact on glycemic control and improved self‐management behaviors. The level of glycemic control and self‐management skills were improved through individual and telephone‐based intervention respectively. Community worker‐led interventions were effective in improvements in diabetes knowledge and self‐care behaviors; however, nurse‐led interventions were effective in glycemic control. Better glycemic control is achieved in hospital settings compared to outpatient settings. HL interventions yielded better improvement in self‐management among people with longer diabetes duration (more than 7 years). It was possible to achieve a large reduction in HbA1c level after a 3‐month intervention in hospital settings. HL‐driven interventions are effective in glycemic and diabetes self‐management outcomes.

## Graphical abstract

- Health literacy (H)L‐driven interventions were effective in controlling glycemic levels and improving diabetes self‐management outcomes.
- The level of glycemic control and self‐management skills were improved better through individual and telephone‐based intervention, respectively, in comparing group intervention.
- Community worker led interventions were effective in improvements in diabetes knowledge and self‐care behaviors; however, nurse‐led interventions were more effective in glycemic control.
- Better glycemic control was achieved in hospital settings compared with outpatient settings.
- HL interventions yielded better improvement in self‐management among people with a longer diabetes duration (more than 7 years) than those with short duration of diabetes.
- It was possible to achieve a significant reduction in HbA1c level after a 3‐month intervention in hospital settings.

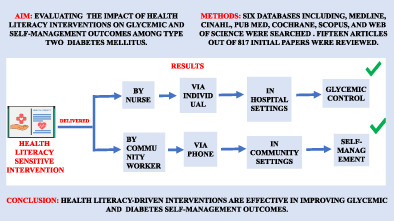

## 摘要

糖尿病给世界范围内的患者和社会带来了日益严重的健康和经济负担。控制血糖是降低2型糖尿病(T2DM)发病率和死亡率的必要条件。自我管理是糖尿病管理的主要手段。健康知识(HL)是自我管理活动的主要动力。本综述旨在评估HL干预对2型糖尿病患者血糖控制和自我管理结果的影响。通过检索MEDLINE、CINAHL、PubMed、Cochrane、Scopus和Web of Science，本综述共纳入1997—2021年发表的15篇英文形式发表的采用HL驱动干预，并监测2型糖尿病患者糖化血红蛋白(HbA_1c_)和自我管理水平的随机对照试验。研究结果表明，健康素养干预对血糖控制和改善自我管理行为产生积极的影响。通过个人干预和电话干预能够提高患者血糖控制水平和自我管理能力。社区工作者主导的干预措施在改善糖尿病知识和自我护理行为方面是有效的；护士主导的干预措施对血糖控制有效。与门诊患者相比，住院患者的血糖控制效果更好。在糖尿病病程较长(超过7年)的人群中，HL干预能更好地改善自我管理。在医院环境中，经过3个月的干预，HbA_1c_水平有可能大幅降低。HL驱动的干预措施对血糖和糖尿病自我管理结果有效。

## 1 INTRODUCTION

Diabetes imposes an increasing health and economic burden on individuals living with it and for their societies worldwide. There were 537 million people living with all types of diabetes in 2021 and this number is predicted to increase to 643 million and 783 million by 2030 and 2045 respectively.^1^ Type 2 diabetes mellitus (T2DM), which accounts for 98.3% of diabetes cases,^2^ is a complex metabolic disorder characterized by insulin resistance and pancreatic beta‐cell dysfunction.^3^ Hyperglycemia caused by T2DM often leads to various microvascular (eg, retinopathy, neuropathy, nephropathy) and macrovascular (coronary artery disease, cerebrovascular disease) complications.^4^ Diabetes is a global health concern that has a significant impact on society and the economy. It results in increased medical expenses, premature death, decreased productivity, and lower quality of life.^5^ According to the American Diabetes Association, the overall expenses associated with diabetes have risen from $245 billion in 2012 to $327 billion in 2017, representing a 26% increase over a five‐year period. Individuals with diabetes have medical expenditures that are roughly twice as high as those without diabetes.^6^ In 2021, diabetes resulted in health expenditures of at least US$966 billion globally, reflecting a 316% increase over the last 15 years.^1^ Complications related to diabetes in the lower extremities are a significant and expanding source of disability across the globe.^7^

In addition to the morbidity of diabetes it is one of the leading causes of mortality worldwide; for instance, globally, there was a 5% increase in premature mortality due to diabetes between 2000 and 2016.^8^ The number of deaths worldwide related to diabetes has risen sharply from 1.5 million in 2012^9^ to 6.7 million in 2021.^1^

Glycemic control is necessary to reduce morbidity and mortality of T2DM. According to a diabetes control and complications trial, normalization glycohemoglobin A1c (HbA1c < 7%) is associated with prevention of diabetes complications such as neuropathy, retinopathy, and nephropathy.^10^ Each 1% reduction in HbA1c decreases the risk of microvascular complications by 37%, deaths related to diabetes by 21% and myocardial infarction by 14%.^11^ Reducing the HbA1c level by 1% is associated with a 13% decrease in diabetes‐related total health care costs.^12^ Glycemic control is also the most important behavioral and therapeutic goal in diabetes care.^13^ Self‐management is the primary tool for managing diabetes^14^ and the aim of diabetes self‐management is to control blood glucose and reduce the risk of diabetes‐related complications.^15^ diabetes self‐management involves a collaborative effort by health care providers and patients in which individuals with diabetes acquire the necessary knowledge and abilities to make behavioral adjustments that help them manage the disease.^16^ Diabetes self‐management focuses on healthy eating, physical activity, monitoring blood sugar, medication adherence, problem‐solving, and healthy coping mechanisms.^17^ Self‐management interventions are associated with improving glycemic control, quality of life, and diabetic complications.^18^

Health literacy (HL) is the primary driver of self‐management activities. HL is the capacity to read, understand, make decisions, and take actions that affect health status.^19^ Low HL has been linked to several negative health outcomes, such as poorer overall health, higher hospitalization and mortality rates, reduced ability to manage chronic illnesses, and increased patient expenses. Individuals with appropriate HL levels are more likely to use available health services and make well‐versed health decisions.^20^ Low HL is a major barrier to the development of self‐management skills.^21^ Further, HL is associated with confidence in self‐managing diabetes.^22^ The low level of HL about the importance of controlling blood glucose is the key barrier to diabetes self‐management.^23^

There is increasing evidence demonstrating a strong association of HL with diabetes knowledge; however, little is known about the effectiveness of HL‐driven interventions specific to T2DM self‐management and glycemic control. This systematic review aims to address this gap by assessing the effectiveness of HL intervention on glycemic control (HbA1c) and T2DM self‐management using randomized controlled trials (RCTs).

## 2 METHODS

### 2.1 Search strategy

To identify HL‐driven T2DM self‐management interventions focusing on glycemic control, we searched six databases including MEDLINE, CINAHL, PubMed, Cochrane, Scopus, and Web of Science. Search terms included “health literacy,” “diabetes mellitus type 2,” “self‐management,” “glucose control,” and “clinical trials” (see Box 1).

BOX 1 **Example of search strategy for review.**

1. “health literacy” or “health education” or “health knowledge” or “health information” or “health understanding”.

2. “diabetes mellitus type 2” or “diabetes type 2”.

3. “self‐management” or “self‐care” or “self‐regulation” or “self‐monitoring”.

4. “glucose control” or “glycemic control” or “sugar control” or “HbA1c”.

5. “clinical trials or randomized controlled trials or controlled clinical trials”.

**FIGURE 1:**
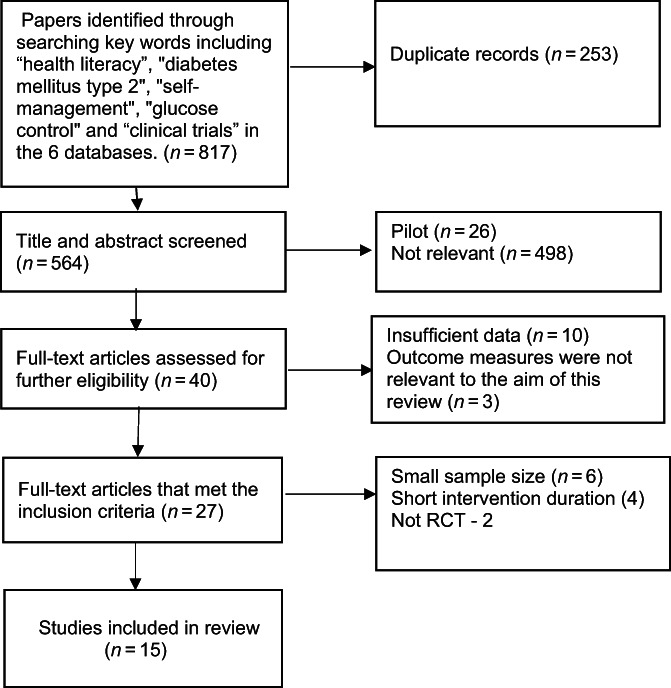
Flow chart of study search and selection of selected papers.

The protocol of this systematic review is registered in PROSPERO (International Prospective Register of Systematic Reviews) (CRD42022348050). The systematic review was performed using the PRISMA (Preferred Reporting Items for Systematic Reviews and Meta‐Analyses) guidelines (see Figure 1). Initial search identified 817 studies through the six databases searched. After excluding the duplications of 564 articles and title and abstract screening, 40 articles were eligible for full‐text screening. The 40 papers were assessed against the inclusion criteria (criteria listed further in the next section) and 15 articles were included in the review.

**FIGURE 2:**
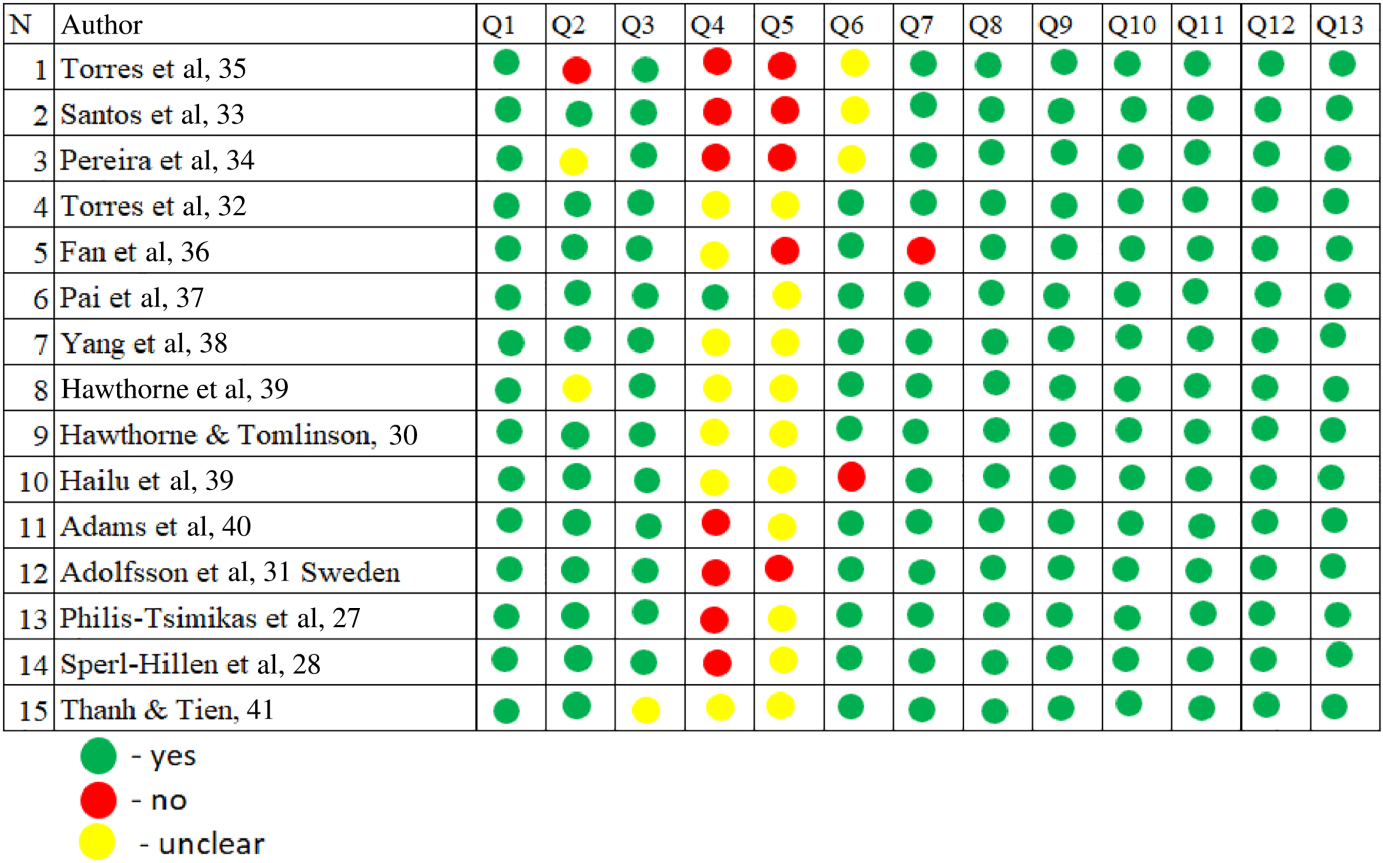
Evaluation of the quality of articles using Joanna Briggs Institute (JBI) tool.

The quality of each study was assessed independently by two authors (Jamila Butayeva, Zubair Ahmed Ratan) using the critical appraisal checklist tool developed by Joanna Briggs Institute (JBI) This tool is the most coherent and sensitive tool for validity, with its focus on congruity.^24^ The JBI critical appraisal tool is a widely accepted method for evaluating the methodological quality of studies.^25^ All the disagreements were resolved by discussion with the senior researcher (Hassan Hosseinzadeh). The JBI score was calculated for each study. A JBI score of 70%–100% was considered as high quality, a score of 69%–50% was considered moderate quality, and <49% was considered low quality. Overall mean score for quality was 80.9% (see Figure 2).

### 2.2 Inclusion and exclusion criteria

Only HL‐driven RCTs published in English and conducted among T2DM patients aged over 18 years were included in this review. Eligible studies had to report both glycemic control and self‐management outcomes and use a HL‐driven intervention. Trials with a minimum sample size of 100 and an intervention duration of >3 months were included. This is because RCTs using smaller samples and shorter intervention delivery time were seen as a threat to the validity and generalizability of research results.^26^

## 3 RESULTS

### 3.1 General study description

**FIGURE 3:**
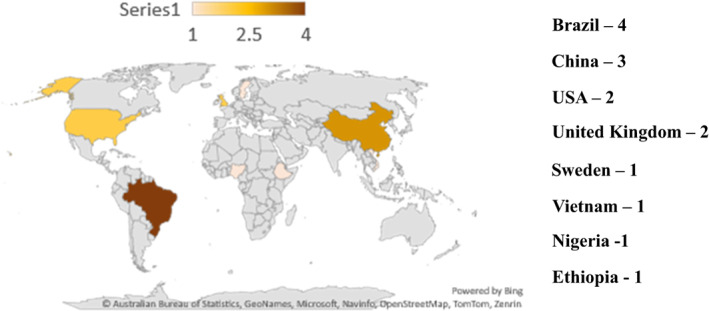
Global map indicating countries where selected studies were conducted.

All of the 15 selected articles were published in English between 1997 and 2021. Five of them were conducted in developed countries including 2 in the United States,^27^, ^28^ 2 in England,^29^, ^30^ 1 in Sweden,^31^ and 10 in developing countries including 4 in Brazil,^32^, ^33^, ^34^, ^35^ 3 in China,^36^, ^37^, ^38^ 1 in Ethiopia,^39^ 1 in Nigeria,^40^ and 1 in Vietnam^41^ (see Figure 3). Three studies were conducted in hospital settings^36^, ^37^, ^41^ and the rest were conducted in outpatient clinics, diabetes clinics, primary health care, or community health centers (see Table 1). From selected articles three were three‐arm RCTs^28^, ^33^, ^34^ and the rest were two‐arm RCTs.

**TABLE 1:** Demographic (age, gender) and intervention (settings, program, length, and follow up time) characteristics.

Author, year, country	Population		Intervention				
	Sample size	Age	Settings	Intervention program	Length оf intervention	Intervention delivery way	Follow‐up
Torres et al, 2018, Brazil^32^	104 (male 26, female 78), control 50, intervention 54	30–70	Outpatient clinic	Diabetes education program by nurse	6 months	Group individual	6 months
Santos et al, 2017, Brazil^33^	238 (male 80, female 158), control 111, intervention 127	30–80	Primary health care centers	Diabetes empowerment program by facilitator and a support professional	12 months	Group, home visits	12 months
Pereira et al, 2021, Brazil^34^	208 (male 84, female 124), control 89, intervention 119	18–79	Basic health units	Diabetes empowerment program by nurse and nutritionist	12 months	Group, telephone	12 months
Torres et al, 2009, Brazil^35^	341 (male 96, female 245), control 171, intervention 170	30–70	Basic health units	Educational program by nurses and nutritionist	12 months	Group education, home visit, telephone	9 months
Fan et al, 2016, China^36^	276 (male 158, female 118), control 138, intervention 138	63 ± 10	Hospital	Diabetes education by nurse	3 months	Group individual	6 months
Pai et al, 2021, China^37^	108 (male 51, female 57), control 55, intervention 53	Age 20 <	Hospital	Technology education program by diabetes educator	7 months	Group	6 months
Yang et al, 2015 China^38^	245 (male 132, female 113), control 116, intervention 121	20–70	Diabetes clinics	Diabetes education program using CM by diabetes educator	12 weeks	Group	12 months
Hawthorne et al, 2001, England^29^	200 (male 95, female 105), control 91, intervention 109	Age 18<	Diabetes center or diabetes mini clinics	Diabetes health education by community worker	6 months	Group	6 months
Hawthorne and Tomlinson, 1997, England^30^	201, (male 93, female 107), control 89, intervention 112	Age 18 <	Diabetes center and general practices	Structured education package by community worker	6 months	Group	6 months
Hailu et al, 2018, Ethiopia^39^	220 (male 148, female 72), control 104, intervention 116	30 years <	Medical center	DSME by nurse	6 months	Group	9 months
Adams et al, 2021, Nigeria^40^	120 (male 46, female 74), contol 60, intervention 60	30–80	Outpatient clinic	DSME by nurse	3 months	Group	3 months
Adolfsson et al, 2007, Sweden^31^	101 (male 52, female 49), control 51, intervention 50	Age 75 <	Primary care centers	Empowerment education by nurse	7 months	Group	12 months
Philips‐Tsimikas et al, 2011, United States^27^	207 (male 61, female 146), control 103, intervention 104	21–75	Community health centers	Peer‐education curriculum by peer educator	3 months	Group	4 months
Sperl‐Hillen et al, 2010, United States^28^	623 (male 315, female 308), control 134, intervention 489	Age 85>	Outpatient clinic	Education programs by nurses and dietitians	77 and 90 days	Group individual	4 months
Thanh et al, 2021, Vietnam^41^	364 (male 165, female 199), control 182, intervention 182	40–80	Hospital	Community education by medical doctors	3 months	Group	3 months

Abbreviations: CM, conversation map; DSME, diabetes self‐management education.

### 3.2 Patients' characteristics

Overall, 3556 participants were included in the selected studies. The proportion of men and women varied across trials but most of the participants (55%) were female. The sample size ranged from 101 to 623 participants. The participants' age ranged between 18 and 85 years old (see Table 1).

### 3.3 Description of interventions

Included studies were heterogeneous in terms of type of intervention, diabetes population, and outcomes assessed. Interventions varied in terms of the type of intervention programs, duration of program, follow‐up period, and intervention providers. Intervention time varied from 3 months^27^, ^28^, ^36^, ^38^, ^40^, ^41^ to 12 months.^33^, ^34^, ^35^ The long follow‐up period of 12 months was performed in four studies,^31^, ^33^, ^34^, ^38^ and 6‐month follow‐up was performed in five studies.^29^, ^30^, ^32^, ^36^, ^37^ In eight studies, interventions were delivered by a nurse,^28^, ^31^, ^32^, ^34^, ^35^, ^36^, ^39^, ^40^ in two studies interventions were delivered by a diabetes educator,^37^, ^38^ in two studies interventions were delivered by a community worker,^29^, ^30^ in one study the intervention was delivered by a support professional,^33^ in one study the intervention was delivered a peer educator,^27^ and in one study the intervention was delivered by a medical doctor.^41^

The most common method of intervention delivery was group education.^27^, ^29^, ^30^, ^31^, ^37^, ^38^, ^39^, ^40^, ^41^ In three studies, a combination of group and individual education,^28^, ^32^, ^36^ group education and telephone calls,^34^ and group education and home visits^33^ were used. In one study, a combination of group education, home visit, and telephone calls was used.^35^

### 3.4 Main findings

The selected studies focused on several outcomes, but we concentrated only on HbA1c and self‐management outcomes (see Table 2).

**TABLE 2:** Main outcomes including HbA1c and self‐management behaviors among the selected studies.

Author, year, country	Study measures	Results			*p* value
		Control	Intervention 1	Intervention 2	
Torres et al,^32^ 2018, Brazil	HbA1c	NA	(individual education) 7.9 ± 1.6	(group education) 7.6 ± 1.4	.002
	Self‐care management	NA	3.6 ± 0.7	3.7 ± 0.5	.641
	Diabetes knowledge	NA	11.1 ± 2.6	10.2 ± 2.0	.017
	Quality of life	NA	112.9 ± 18.1	107.6 ± 18	.825
Santos et al, 2017, Brazil^33^	HbA1c	7.4	(home visit) 7.0	(group education) 7.1	.0000 (group) .9900 (home visit)
	Diabetes Empowerment Scale	4	4.25	4.13	.0000 (group) .0000 (home visit)
	Self‐care adherence	3	5	4.05	.0001 (group) .0001 (home visit)
Pereira et al, 2021, Brazil^34^	HbA1c	8.3	(telephone education) 7.3	(group education) 6.9	.003 (group) < .001 (telephone)
	Self‐care practices	2.5	4.2 ± 1.1	3.4 ± 1.1	.786 (group) < .001 (telephone)
	Diabetes empowerment	30	34.0	32.2 ± 3.3	.211 (group) < .001 (telephone)
Torres et al, 2009, Brazil^35^	HbA1c	8.29	7.93	NA	< .05
Fan et al, 2016, China^36^	HbA1c	NA	(individual education) 6.21 ± 0.56	(group education) 6.95 ± 3.12	.027
Pai et al, 2021, China^37^	HbA1c	NA	6.70 ± 0.74	7.307 ± 1.31	< .05
	Perceived Diabetes Self‐Management Scale	NA	31.67 ± 5.07	27.59 ± 5.16	< .05
Yang et al, 2015, China^38^	HbA1c	9.77	7.55	NA	< .01
	Self‐care behavior	NA	NA	NA	NA
Hawthorne et al, 2001, England^29^	HbA1c	8.4	7.7	NA	< .01
	Diabetes knowledge	NA	NA	NA	.05
Hawthorne et al, 1997, England^30^	HbA1c	8.64	8.3	NA	NA
	Diabetes knowledge	NA	NA	NA	NA
Hailu et al, 2018, Ethiopia^39^	HbA1c	Reduction by 2.57%	Reduction by 2.88%	NA	.208
Adams, 2021, Nigeria^40^	HbA1c	6.8 1 ± 0.3	5.6 ± 1.0	NA	< .001
Adolfsson et al, 2007, Sweden^31^	HbA1c	7.4	7.3	NA	NA
	Self‐efficacy	4.0	9.8	NA	.272
	Diabetes knowledge	5.1	14.8	NA	.012
	Satisfaction with daily life	0.0	2.5	NA	.588
Philips‐Tsimikas, 2011, United States^27^	HbA1c	NA	(individual education) 9.1	(group education) 9.7	.01
Sperl‐Hillen et al, 2010, United States^28^	HbA1c	7.77	7.52	7.66	< .001 (group) < .001 (individual)
	Food score	12.37	12.99	12.89	.006 (group) < .001 (individual)
	Physical activity	121.37	145.24	122.37	.64 (group) < .08 (individual)
Thanh et al, 2021, Vietnam^41^	HbA1c	8.15 ± 1.8	7.56 ± 1.64%	NA	.001
	Diabetes knowledge	8.57 ± 2.86	10.52 ± 2.08	NA	< .001

Abbreviation: HbA1c, glycohemoglobin A1c.

#### 3.4.1 Glycemic control (HbA1c)

All studies evaluated the effect of a HL‐driven intervention on HbA1c; 14 studies showed that HL had a significant and positive impact on HbA1c control and 1 study did not find any significant changes in HbA1c level after a HL intervention program.^31^ The largest reduction in HbA1c level (3.4%) was achieved after a 3‐month nurse‐led individual intervention with a 6‐month follow‐up in a hospital setting.^36^ Diabetes education intervention delivered by a nurse in an outpatient setting was more effective in reducing HbA1c (1.5% reduction) among more educated participants.^32^ Female and educated participants as well as those who participated in more educational sessions had better results in glycemic control in outpatient settings.^27^, ^30^, ^38^ Similarly, educated women and participants who had higher levels of HbA1c at the baseline were more likely to have better improvements in HbA1c in outpatient settings.^28^, ^29^, ^39^ Participants from urban area and those with low body mass index (BMI) levels demonstrated substantially lower outcomes in HbA1c level in outpatient settings.^27^, ^32^, ^35^, ^39^ Surprisingly, participants who did not have any occupation were more likely to have a better glycemic control after long intervention periods of 12 months^33^, ^34^, ^35^ and 7 months.^37^ HL‐driven interventions were also more effective in HbA1c control among people who had comorbidities and low levels of alcohol consumption.^28^, ^34^, ^37^, ^41^

#### 3.4.2 Diabetes self—management

Self‐management outcomes were evaluated in nine studies.^28^, ^30^, ^31^, ^33^, ^34^, ^35^, ^37^, ^38^, ^41^ Of them, five evaluated diabetes knowledge,^29^, ^30^, ^31^, ^32^, ^41^ and all of them found a positive correlation between HL interventions and diabetes knowledge. Interventions delivered by community workers compared to those delivered other providers were more likely to improve diabetes knowledge among educated women.^29^ Nurse‐led intervention using group education with a 6‐month follow‐up delivered in outpatient settings resulted in a greater increased diabetes knowledge compared to tailored education interventions.^35^

Nine articles evaluated the impact of HL on self‐management.^28^, ^29^, ^30^, ^31^, ^32^, ^33^, ^34^, ^37^, ^38^ Only two of them did not find any association between HL with self‐management behaviors, quality of life, and satisfaction with daily life.^31^, ^32^ Patients with longer disease duration and those received an intervention through home visits achieved better self‐care adherence.^28^, ^29^, ^33^, ^38^ A telephone intervention with a 12‐month diabetes empowerment program delivered by a nurse was more likely to result in a better self‐care practice than group education.^34^

Regardless of the type and setting of the interventions, better HL improved self‐care behaviors including being physically active, problem‐solving skills, healthy coping strategies, quality of life, satisfaction with daily life, and managing hyperglycemia in the intervention groups compared to control groups.^28^, ^29^, ^30^, ^38^

Educated women showed better results in managing hyperglycemia and regular glucose checks after 6‐month community worker‐delivered intervention in diabetes clinics compared to noneducated women.^29^, ^30^ Physical activity level and adherence to a healthy diet were more likely to increase in the intervention group compared to the control group after interventions delivered by nurses and dietitians in outpatient settings.^28^, ^32^

## 4 DISCUSSION

Our systematic review of 15 included articles showed that HL intervention was effective in glycemic control and improving diabetes knowledge and self‐management skills among diabetes patients. Fourteen out of 15 included RCTs showed that HL interventions resulted in significant improvements in HbA1c levels. However, HL‐driven interventions delivered through individual education at hospital settings were more effective in decreasing uncontrolled levels of HbA1c compared to HL‐driven interventions delivered using group education. This might be due to tailored education and physical proximity to health care services at the hospitals, which might facilitate the impact of HL interventions.^42^ Literature suggests that tailored education based on patient needs motivated them to adhere to life adjustment measures.^43^ In line with a recent systematic review,^44^ our findings showed that HL interventions resulted in more improvements in HbA1c levels among patients with suboptimal glucose levels where the baseline mean of HbA1c was >9%.^28^, ^29^, ^39^ This finding suggests that T2DM patients with poorly controlled HbA1c levels are more likely to benefit from HL intervention.^45^ There was a positive relationship between the amount of time participants spent on education sessions during HL education intervention and improvement in glycemic control. In other words, in line with a recent systematic review,^46^ participants who engaged more with HL interventions achieved more improvements in glycemic control. Our findings showed that nurse‐led interventions were associated with better glycemic control^28^, ^32^, ^34^, ^35^, ^36^, ^39^, ^40^ which may be explained by the fact that practice nurses are capable of dealing with complicated health problems and can give information and support to patients and their families efficiently, which are essential in empowering patients to adopt new behavior.^47^ Our review highlights that after participating in a 3‐month peer‐education program, individuals in urban areas are more likely to achieve better glycemic control compared to those in rural areas.^39^ This might be explained by the literature suggesting that people residing in rural areas tend to perceive ill health and mortality as natural phenomena, whereas individuals living in urban areas are less accepting of ill health and more likely to seek health care advice.^48^ There was also a positive linear correlation between BMI and HbA1c levels and participants with higher BMI index had higher level of HbA1c. Participants with low BMI level had lower HbA1c level after interventions in some studies.^27^, ^32^ This is consistent with previous literature, which showed that BMI is a significant predictor of poor HbA1c control^49^ and the increase in BMI levels leads to rising in insulin levels resulting in increasing HbA1c levels.^50^

Five articles that examined diabetes knowledge showed significant improvements in diabetes knowledge after HL interventions.^29^, ^30^, ^31^, ^32^, ^41^ HL interventions resulted in a greater impartment in diabetes knowledge when it was delivered by community workers compared other health care providers, which is in line with the findings of a recent systematic review showing that HL interventions were positively correlated with diabetes knowledge and glycemic control.^51^ This might be explained by the fact that community workers are more likely to share similar cultural, linguistic, and socioeconomic backgrounds with patients, which are essential for providing interventions meeting real‐world needs.^52^ Our findings also showed that more educated women were more likely to achieve greater impartments in diabetes knowledge when HL interventions were delivered by community workers compared to noneducated women. Similarly, a recent study showed that patients with high literacy level were 1.85 times more likely to gain diabetes knowledge required to manage their diabetes compared to their less educated counterparts.^53^ This might be because highly educated people are most likely to understand the salience of health information and are more capable to put new information in practice.^54^

Seven out of nine RCTs that assessed diabetes self‐management found that HL interventions were linked to improved self‐management outcomes^28^, ^29^, ^30^, ^33^, ^34^, ^37^, ^38^ such as physical activity, healthy diet, diabetes knowledge, problem‐solving, and quality of life. We found that that telephone‐based HL interventions among patients with a longer diabetes duration were more likely to yield positive improvements in self‐management behaviors especially among less educated participants compared to face‐to face group education.^33^, ^34^ This might be because telephone‐based interventions may aid less educated people to understand the context of education and give them more chances to ask questions and seek support. Surprisingly, intervention delivered through home visits among patients who had longer diabetes duration were effective in improving self‐management outcomes.^28^, ^29^, ^33^, ^38^ According to literature home visits allow health professionals to see patients' living conditions and involve family members and caregivers in health education, which are critical in gaining better improvements in behavior change interventions.^55^ The reason for the patients with a longer diabetes duration responding well to HL interventions might be because they are more likely to experience the consequences of uncontrolled diabetes, which might motivate them to engage well in HL interventions.

## 5 LIMITATIONS

This study offers invaluable information about the impacts of HL intervention on glycemic control and diabetes self‐management outcomes; however, it has some limitations. Only studies published in English were included in this review, which might lead to the exclusion of high‐quality studies published in other languages. Most of the selected studies were conducted in developed countries, which may limit the generalization of the findings of this review to patients living in developing countries. The heterogeneity of study settings, intervention strategies used, and study participants' socioeconomic characteristics make generalization of the findings to similar populations difficult. A lack of information about the professional background of those who delivered the interventions could affect the quality of the information delivered during the interventions. Using different outcome measures and tools made analysis difficult. The study outcomes were presented for less than 1 year so long‐term effects could not be examined. Only RCTs were included in this review, which might limit the generalizability of these findings to real‐world settings.

## 6 CONCLUSION

Findings from our review of 15 articles suggests group and telephone‐based HL interventions and interventions delivered by nurses and/or community workers and interventions in hospital settings yield promising outcomes in glycemic control and self‐management, even after a short duration. Overall, HL‐driven interventions are effective in glycemic and diabetes self‐management outcomes.

## AUTHOR CONTRIBUTIONS

Hassan Hosseinzadeh: Study design, methodology, data analysis, assessing the quality of the selected papers, reviewing the paper, and final approval. Jamila Butayeva: literature searching, methodology, data analysis, assessing the quality of selected articles, and drafting and finalizing the paper. Zubair Ahmed Ratan: Data analysis, reviewing the paper, and assessing the quality of articles. Sue Downie: data analysis and reviewing the paper. The final version of the paper was approved by all of the authors.

## FUNDING INFORMATION

This review did not receive any funding.

## DISCLOSURE

The authors have nothing to disclose.
